# Association of Sleep Duration, Sleep Quality and Shift-Work Schedule in Relation to Hypertension Prevalence in Chinese Adult Males: A Cross-Sectional Survey

**DOI:** 10.3390/ijerph14020210

**Published:** 2017-02-21

**Authors:** Kai Lu, Jia Chen, Li Wang, Changying Wang, Rongjing Ding, Shouling Wu, Dayi Hu

**Affiliations:** 1 Department of Cardiology, The First Affiliated Hospital of Chongqing Medical University, Chongqing 400042, China; lukai2013@foxmail.com; 2 Department of Clinical Nutrition, The First Affiliated Hospital of Chongqing Medical University, Chongqing 400042, China; drchenjia@foxmail.com; 3 Department of Cardiology, Yong Chuan Hospital, Chongqing Medical University, Chongqing 400042, China; wangli514@sina.cn; 4 Department of Cardiology, Tangdu Hospital, Fourth Military Medical University, Xi’an 710038, China; wangchangying04@163.com; 5 Heart Center, Peking University People’s Hospital, Beijing 100044, China; rongjingding@163.com; 6 Kailuan General Hospital, Hebei United University, Tangshan 100816, China; drwushouling@163.com

**Keywords:** sleep duration, sleep quality, shift-work schedule, hypertension

## Abstract

*Background:* Previous studies indicated that measurement of sleep only by duration and quality may be biased. This study aimed to investigate the interactive association of self-reported sleep duration, quality and shift-work schedule with hypertension prevalence in Chinese adult males. *Methods:* A total of 4519 Chinese adult males (≥18 years) were enrolled into the cross-sectional survey. Sleep attributes were measured from the responses to the standard Pittsburgh Sleep Quality Index and relevant questions in a structured questionnaire survey. The association of sleep duration, quality and shift-work schedule with hypertension prevalence was analyzed using multivariate logistic regression, considering the interaction between them or not. *Results:* Taking the potential interaction of the three aspects of sleep into consideration, only short sleep duration combined with poor sleep quality was found to be related to hypertension prevalence in Chinese adult males (odds ratio (OR): 1.74, 95% confidence interval (CI): 1.31–2.31), which could be modified by occasional and frequent shift-work schedule (OR: 1.43, 95% CI: 1.05–1.95; OR: 1.97, 95% CI: 1.40–2.79). *Conclusions:* Short sleep duration was not associated with the prevalence of hypertension in Chinese adult males unless poor sleep quality exists, which could be further modified by shift-work schedule. Assessment of sleep by measuring sleep duration only was not sufficient when exploring the association of sleep with hypertension.

## 1. Introduction

The association of hypertension in relation to sleep disturbance has been raising the concerns of cardiologists in recent years and the association of sleep duration with hypertension has been studied quite extensively. Most of those studies indicated that short sleep duration was a risk factor for the development of hypertension, but a few well-designed studies denied this association [1,2,3,4,5,6,7]. It is necessary to explore the potential reasons for the conflicts.

Sleep has other aspects besides the qualitative one. Two studies took the both qualitative and quantitative aspects of sleep into consideration when exploring the potential relationship between sleep and hypertension, and the results demonstrated that short sleep duration failed to influence the prevalence or incidence of hypertension unless it was combined with other sleep disturbances, which suggested that it was not enough to evaluate sleep by measuring sleep duration only [2,8] It is well documented that poor sleep quality is associated with the prevalence of obesity, metabolic syndrome and glucose metabolism [9,10,11], which share many common pathways with the development of hypertension. In addition, accumulating evidence shows that the disturbance of sleep circadian rhythm, which is often researched in shift workers, is a potential risk factor for a wide range of cardiovascular or metabolic disorders [12,13,14,15,16,17]. It has been reported that shift work is associated with prevalence of hypertension, but this is still inconclusive [18,19,20,21,22,23]. Therefore, we assumed that both sleep quality and shift-work schedule are also involved in the development of hypertension and they should be evaluated when exploring the possible link between sleep and hypertension.

There is a common limitation of previous studies regarding sleep and hypertension: the studies did not examine the separate role of various sleep aspects in the development of hypertension. Considering the fact that various sleep disturbances often concurrently occur, it is difficult to tell the exact contribution of each sleep aspect to hypertension and the result may be biased. For example, insomnia patients usually suffer sleep quality and sleep circadian rhythm problems besides short sleep duration. There is a high probability that not adjusting the confounders of sleep quality and sleep circadian rhythm may bias the results concerning the association between sleep duration and hypertension and lead to conflicting results.

Based on what is mentioned above, we aimed to elucidate the separate and combined effects of the three aspects of sleep, i.e., sleep quality, sleep duration and shift-work schedule, on hypertension prevalence in Chinese males in this cross-sectional study.

## 2. Methods

### 2.1. Study Design and Population

This study was a cross-sectional survey conducted from September to December 2013 in communities of Fangezhuang, Tangshan, Lvjiatuo and Qianjiaying, which were all functional and comprehensive communities owned and managed by the Kailuan Group in Tangshan City, Hebei Province in north China. The sample population was selected randomly from residences of the four communities aged 18 or older. The main exclusion criteria included: diagnosed or suspected secondary hypertension; severe chronic heart failure; severe liver dysfunction; end-stage renal disease; advanced cancer; previous diagnosis of obstructive sleep apnea syndrome (OSAS) or restless legs syndrome (RLS); and those who were unable to cooperate with physical examination or interview due to mental disorder or physical disability. Sleep circadian rhythm disturbance was mainly evaluated by the frequency of shift work. Female participants were not enrolled in the current study because most of them were office workers on regular daytime shift and thus it was not possible to evaluate the association of sleep circadian rhythm with hypertension for them. In addition, those who changed jobs with different shift-work schedules during the past 12 months were also excluded from the final analysis (*n* = 73). Anthropometric measurements, blood tests and astructured questionnaire survey were administered to each subject after the enrollment.

### 2.2. Measurement

#### 2.2.1. Anthropometric Measurements

Qualified physicians and nurses were trained on the standard study protocols before the survey was initiated. Height and weight was measured to the nearest 0.1 cm and 0.1 kg, respectively, when the participants stood upright and barefoot in light clothes. Two separate measurements of height and weight were recorded for each participant and averaged for analysis. Body mass index (BMI) was calculated as the ratio of weight to height squared (kg/m^2^).

Blood pressure was measured two times with a five-minute interval after a resting period of 10 min in a seated position. Standard mercury sphygmomanometers (Yuyue, China) were used for the measurement of blood pressure. Average of two measurements was recorded as the final blood pressure. However, when the systolic or diastolic pressures exhibited a difference greater than 5 mmHg, a third measurement was necessary and the final blood pressure value was recorded as the average of the three measurements. Hypertension was defined in accordance to the Seventh Report of the Joint National Committee on Prevention, Detection, Evaluation, and Treatment of High Blood Pressurein the current study [24], i.e., SBP (systolic blood pressure) ≥ 140 mmHg and (or) DBP (diastolic blood pressure) ≥ 90 mmHg or current antihypertensive medication.

#### 2.2.2. Laboratory Measurement

Participants were asked to fast overnight before venous blood sample collection. Blood was collected from antecubital veins and centrifuged at 3000 rpm for 10 min at room temperature. All blood samples were tested at the central laboratory of Kailuan General Hospital using automatic biochemical analyzers (Hitachi 717, Tokyo, Japan) within four hours for concentrations of total triglyceride (TG), total cholesterol (TC) and fasting blood glucose (FBG). Kits were provided by the Biology Institute of North China (Xining, China).

#### 2.2.3. Questionnaire Survey

A structured questionnaire survey was administered to each participant face to face on paper to obtain the following information: age, status of smoking and drinking, physical activity, salt intake, educational level, family income and profile of sleep. Status of smoking and drinking was evaluated from self-reported information and was divided into “never”, “former” and “current”. Physical activity was evaluated from responses to questions about type and frequency of physical exercise at work and during leisure time and was categorized into “active” (≥150 min/week aerobic exercise such as jogging, swimming, climbing, etc.) and “inactive”. Salt intake was evaluated from responses to the questions about the amount of salt consumed in the last month and it was divided into “low salt” (≤6 g/day), “medium salt” (7–11 g/day) and “high salt” (≥12 g/day). Educational level was classified into “primacy/illiteracy”, “middle school” and “university/college”. The average monthly income of each family member was reported as “<¥600”, “¥600 to ¥1000”, and “≥¥1000”. Sleep quality was evaluated using the standard Pittsburgh Sleep Quality Index (PSQI), which is a widely used measure of sleep quality. According the study of Tsai et al., the Chinese version of the PSQI has good overall reliability (*r* = 0.82–0.83) and test-retest reliability (*r* = 0.77–0.85) [25]. Sleep quality was assessedby the PSQI total score and classified into “good” (PSQI score ≤ 3), “moderate” (3 < PSQI score ≤ 5), and “poor” (PSQI score > 5). Sleep duration was evaluated from responses to relevant questions about the actual sleep duration every day in the past month and was classified into “short” (<7 h), “normal” (7 h ≤ sleep duration ≤ 8h) and “long” (>8 h) based on previous reports [26,27,28] and the distribution characteristics of our data. Participants were also asked to report their work shift they were on in the past 12 months. In Kailuan Group, three kinds of work shift systems are adopted for employees with different jobs, i.e., regular daytime shift, daytime shift at most times accompanied by occasional on-call night shift, and regular three daily shifts. For those on three daily shifts, they are on and off duty alternatively every eight hours, i.e., from 8:00 a.m. to 4:00 p.m., from 4:00 p.m. to 12:00 p.m. and from 12:00 p.m. to 8:00 a.m. In the current study, work shift covering the period from 12:00 p.m. to 8:00 a.m. was defined as shift-work schedule which was categorized into “never” (never on night shift), “occasional” (on night shift no more than once per week on average) and “frequent” (on night shift more than once per week on average) according to the shift work. 

### 2.3. Statistics

Continuous variables were presented as means ± standard deviations (SD) and categorical variables were presented as frequencies and proportions. In the descriptive analysis, the basic characteristics of the enrolled participants with or without hypertension were presented. Hypertension prevalence was presented according to sleep quality, sleep duration and shift-work schedule. Continuous variables were compared with one-way ANOVA and categorical variables were compared with χ^2^ test. For the analysis of the association of sleep quality, sleep duration, shift-work schedule with hypertension prevalence, univariate logistic regression analysis was first used and then age was adjusted for (adjusted OR ^1^). On this basis, BMI, TC, TG, FBG, physical activity, smoking, drinking, salt intake, educational level and family income were further adjusted for (adjusted OR ^2^). To investigate the separate and combined effects of the three sleep attributes on hypertension prevalence, 11 groups of participants were established by different combinations of the following four sleep elements: short sleep duration (+) or not (−), poor sleep quality (+) or not (−), occasional shift-work schedule (+) or not (−), and frequent shift-work schedule (+) or not (−). Specifically, groups of moderate and poor sleep quality were merged here, considering the number of participants in those groups is too small to finish the following analysis. The cut-off value to judge good or poor sleep quality was PSQI score of 3. Unadjusted and adjusted odds ratio of each group for prevalence of hypertension was also calculated using logistic regression analysis. For all the comparisons, the level of statistical significance was set at *p* < 0.05. SPSS 19.0 was used for the statistical analysis (IBM, North Castle, NY, USA).

### 2.4. Ethical Statement

All participants provided written informed consent and their privacy rights were observed. The study was conducted in accordance with the Declaration of Helsinki, and the protocol was approved by the Ethics Committee of the Kailuan General Hospital (approval code is 2013(5)).

## 3. Results

### 3.1. The Basic Characteristics of Enrolled Participants

A total of 6817 residents were invited into the current survey and we excluded 191 (3.8%) with secondary hypertension, 251 (4.9%) with OSAS, 43 (0.9%) with RLS and 31 (0.6%) with data missing or incomplete at baseline. Missing data were all regarding the assessment of sleep quality due to damaged of questionnaires. Analysis was confirmed to the remaining 4519 subjects. The overall prevalence of hypertension was 27.2% (1231 cases) in the finally enrolled participants. The basic characteristics of subjects with or without hypertension were presented in Table 1. Hypertensive subjects tended to be older, drinkers, smokers, inactive physical exercisers, less education, members of lower family income, take higher salt and have a higher average BMI, WC, TG, and FBG, while TG was higher in normotensive participants. Compared with those with normotension, hypertensive participants had poorer sleep quality (4.26 ± 3.17 vs. 3.55 ± 2.90, *p* < 0.01) and shorter sleep duration (6.74 ± 1.20 h vs. 6.98 ± 1.48 h, *p* < 0.01). As to shift-work schedule, participants with hypertension had lower prevalence of “occasional shift-work schedule” (27.7% vs. 34.2%, *p* < 0.01) and higher prevalence of “frequent shift-work schedule” (24.9% vs. 19.9%, *p* < 0.01) than those without.

### 3.2. Hypertension Prevalence According to Sleep Quality, Sleep Duration and Shift-Work Schedule

**Figure 1:**
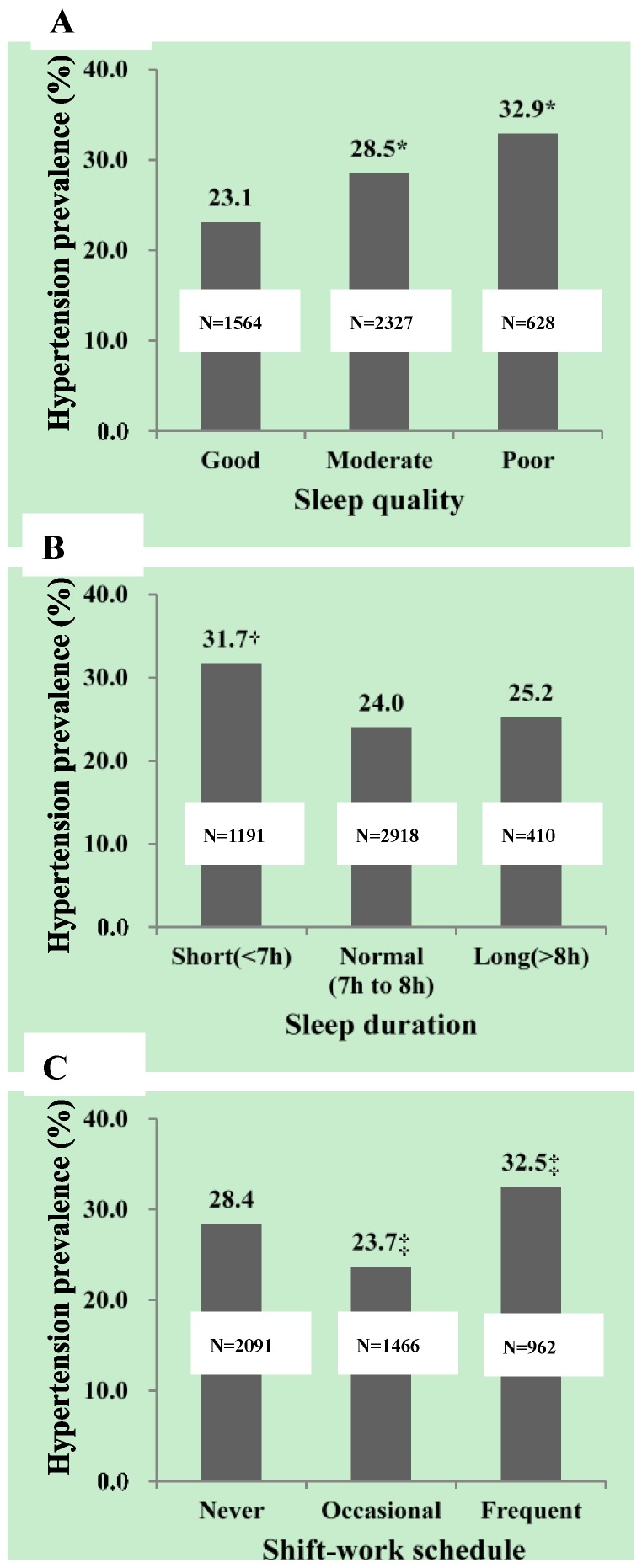
Hypertension prevalence in enrolled subjects according to sleep quality, sleep duration and shift-work schedule: (**A**) hypertension prevalence in participants with different sleep quality; (**B**) hypertension prevalence in participants with different sleep duration; and (**C**) hypertension prevalence in participants with different frequencies of shift-work schedule. The number in the middle of each column indicates the number of subjects the column stand for. * *p* < 0.05 vs. the “good” group; **^†^** *p* < 0.05 vs. the “normal” group; **^‡^** *p* < 0.05 vs. the “never” group.

Figure 1 presents the hypertension prevalence in participants with different sleep quality (Figure 1A), sleep duration (Figure 1B) and shift-work schedule (Figure 1C). In Chinese adult males enrolled in the current study, hypertension prevalence increased from 23.1% to 32.9% (*p* < 0.01) with sleep quality getting worse from “good” to “poor”. Prevalence of hypertension was significantly higher in participants with short sleep duration than those with normal sleep duration (31.7% vs. 24.0%, *p* < 0.01) while no significant difference was found between those with normal and long sleep duration (24.0% vs. 25.2%, *p* > 0.01). In comparison to participants with never shift-work schedule, hypertension prevalence was significantly lower in those with occasional shift-work schedule (28.4% vs. 23.7%, *p* < 0.01) and significantly higher in those with frequent shift-work schedule (28.4% vs. 32.5%, *p* < 0.01).

### 3.3. The Association of Sleep Quality, Sleep Duration and Shift-Work Schedulewith Hypertension Prevalence

Table 2 summarizes the odds ratios (OR) of sleep quality, sleep duration and shift-work schedule for hypertension in Chinese adult males. Sleep quality (global PSQI score) was significantly related to prevalence of hypertension (OR = 1.07, 95% CI, 1.04–1.10) after adjusting for the basic cardiovascular characteristics. With good sleep quality as the reference, moderate and poor sleep quality were significantly related to increased prevalence of hypertension (OR = 1.22, 95% CI, 1.03–1.45; OR = 1.81, 95% CI, 1.44–2.29). As shown in Table 2, increase of sleep duration could bring down the risk for hypertension (OR = 0.87, 95% CI, 0.81–0.92). In comparison to people with normal sleep duration, those with short sleep duration had a higher risk for hypertension (OR = 1.33, 95% CI, 1.13–1.56). However, no significant association was found between long and normal sleep duration in the enrolled subjects. In comparison to never shift-work schedule, both occasional and frequent shift-work schedule were associated with prevalence of hypertension and their corresponding odds ratios are 0.81 (95% CI, 0.68–0.96) and 1.20 (95% CI, 1.01–1.44), respectively.

### 3.4. Interaction of Sleep Duration, Sleep Quality and Shift-Work Scheduleon Hypertension Prevalence

To further investigate the separate and combined association of the three sleep attributes with hypertension prevalence, enrolled participants were divided into 11 groups with different combinations of sleep quality, sleep duration and shift-work schedule, as shown in Table 3. None of the respective associations of poor sleep quality, short sleep duration or frequent shift-work schedule with the prevalence of hypertension reached statistical significance and only occasional shift-work schedule was found to be associated with decreased hypertension prevalence with an odds ratio of 0.67 (95% CI, 0.50–0.89). Short sleep duration had to be combined with poor sleep quality to exert an effect on hypertension (OR = 1.74, 95% CI, 1.31–2.31). This could be modified by occasional and frequent shift-work schedule and their corresponding odds ratios are 1.43 (95% CI, 1.05–1.95) and 1.97 (95% CI, 1.40–2.79), respectively.

## 4. Discussion

The relationship between sleep and hypertension prevalence in China adults has not been fully elucidated previously. In this cross-sectional survey, we explored the potential association of sleep quality, sleep duration and shift-work schedule with the prevalence of hypertension in Chinese males. Our data revealed that short sleep duration, poor sleep quality and frequent shift-work schedule were associated with the increased hypertension prevalence while occasional shift-work schedule was found to be associated with the decreased prevalence of hypertension if the potential interaction between them was disregarded. However, all of those associations failed to reach statistical significance with the exception of occasional shift-work schedule when taking the interaction of them into consideration and short sleep duration had to be combined with poor sleep quality to be related to hypertension in that case.

The association of short sleep duration with hypertension has been explored by many cross-sectional and longitudinal studies [29]. However, obvious conflicting results could be found among those reports. We believed that failing to control the potential confounding effects of other aspects of sleep, such as sleep quality, may be one important reason that leaded to the poor consistence of those previous results. Evidences from cross-sectional and prospective studies are accumulating that short sleep duration alone is not significantly related to the development of hypertension unless the presentation of poor sleep quality or other sleep disorders [2,8]. Besides, Bruno RM et al. reported that poor sleep quality was directly associated with resistant hypertension [30].

The results of previous studies concerning the association of hypertension with shift-work were inconsistent. Three studies in Japanese male workers showed higher prevalence and incidence of hypertension in shift workers [21,22,23]. However, the results of Finnish Twin Cohort and one nursing cohort in Brazil population did not show the association of shift work with the increase in blood pressure level and the incidence of hypertension [19,20]. In the present study, we examined the link of frequent shift work and occasional shift work with hypertension separately and got an interesting result. Frequent shift-work schedule was observed to be relevant to the increased prevalence of hypertension which was in line with our expectation and consistent with some previous reports. Nevertheless, the result that occasional shift-work schedule was associated with reduced hypertension prevalence even after the adjustment of other sleep aspects was a little surprising to us. Changes of activities of daily life, such as habitual diet and physical activities, as well as neuroendocrine functional disorders caused by circadian disruption are believed to mediate the increased cardiovascular risk for workers with sleep disturbance [9,31,32,33]. In the current study, we did not find a significant difference as to the physical activities between participants on regular daytime duty and occasional night duty (31.3% vs. 31.9%, *p* > 0.05). Unfortunately, habitual diets and the levels of endocrine hormone or factors were neither measured in the current study nor reported previously. Thus, the potential mechanism under such a paradox association of shift work schedule and hypertension is still unclear to us.

The most important finding of the current study was the association of the three attributes of sleep, i.e., sleep duration, sleep quality and shift-work schedule, with hypertension seemed to be additive. Measuring sleep duration only was insufficient when investigating the association of sleep with hypertension. Neglecting the potential confounding effects of other aspects of sleep such as sleep quality and circadian rhythm on hypertension may be one important cause of the conflicting results concerning sleep duration and hypertension. Our result was corroborated by some previous studies aiming to explore the interaction of sleep duration and sleep quality on impaired fasting glucose and cardiovascular events which also indicated an additive effect of them [11,34,35]. However, our work was the first report on the interaction of sleep quality, duration and circadian rhythm on hypertension in Chinese population.

Our findings were based on a cross-sectional study and there are several limitations about the observations. First, the cross-sectional design could not provide causal relationship between characteristics of sleep and hypertension prevalence. Secondly, there were no standard cut-off values to judge normal or abnormal sleep time, good or poor sleep quality and occasional or frequent sleep circadian rhythm disturbance. The cut-off values used in this study were based on the previous reports and the basic features of our data. Thirdly, habitual diet is one important confounding factor for the association of sleep disorders with hypertension, but it was not measured and controlled in the present study because accurate and convenient tools for measurement of habitual diet suitable for a large population were not available to us. Fourthly, we did not consider the difference in sleep duration between weekday and weekend. Although most of those participants work six days a week and people working on regular three daily shifts do not stop working on weekend, failing to notice this difference will inevitably lead to a bias on the results. Fifthly, although we ruled out all the participants with previous diagnosis of OSAS and RLS at the time of enrollment, there is the possibility of co-morbid primary sleep disorders such as OSAS and RLS in our data because we did not screen those sleep disorders due to the limited funds and human resources. The impacts of those undiagnosed sleep disorders on the results are unpredictable. Sixthly, since all the participants enrolled in the present study came from north China and we did not adjust for other living habits, we hold a cautious attitude to extrapolate the conclusion to the whole country.

## 5. Conclusions

Despite the above limitations, this cross-sectional study demonstrates an additive association of sleep duration, sleep quality and shift-work schedule with hypertension in Chinese adult males. Specifically, short sleep duration must be combined with poor sleep quality to have a significant association with hypertension, and shift-work schedule could modify this association. This study also indicates that measurement of sleep should be done comprehensively, and only measuring sleep duration may bias the result when investigating sleep and hypertension.

## Figures and Tables

**Table 1:** The basic characteristics of participants with or without hypertension.

Potential Risk Factor for Hypertension	Hypertension (*n* = 1231)	Non-Hypertension (*n* = 3288)	*p*	Total (*n* = 4519)
Age (year)	48.13 ± 6.56	46.36 ± 9.60	<0.01	46.84 ± 8.84
BMI (kg/m^2^)	25.89 ± 3.60	25.07 ± 3.60	<0.01	25.30 ± 3.62
Overweight (28 > BMI ≥ 24) (*n*, %)	558 (45.3)	1413 (43.0)	<0.01	1971 (43.6)
Obesity (BMI ≥ 28) (*n*, %)	361 (29.3)	490 (14.9)	-	851 (18.8)
SBP (mmHg)	134.59 ± 14.47	128.03 ± 14.25	<0.01	129.85 ± 14.55
DBP (mmHg)	89.17 ± 9.94	84.36 ± 9.28	<0.01	85.66 ± 9.70
TC (mmol/L)	4.95 ± 0.93	4.86 ± 0.95	<0.01	4.88 ± 0.94
TG (mmol/L)	1.88 ± 1.81	2.10 ± 2.10	<0.01	1.94 ± 1.92
FBG (mmol/L)	5.59 ± 1.68	5.41 ± 1.41	<0.01	5.46 ± 1.49
Physical activity (*n*, %)			<0.01	
Active	351 (28.5)	1134 (34.5)	-	1396 (32.9)
Inactive	880 (71.5)	2154 (65.5)	-	3034 (67.1)
Smoking (*n*, %)			<0.01	
Never	392 (31.8)	1453 (44.2)	-	1845 (40.8)
Former	118 (9.6)	227 (6.9)	-	619 (7.6)
Current	722 (58.7)	1608 (48.9)	-	2342 (51.6)
Drinking (*n*, %)			<0.01	
Never	383 (31.1)	1302 (39.6)	-	1685 (37.3)
Former	76 (6.2)	191 (5.8)	-	267 (5.9)
Current	772 (62.7)	1795 (54.6)	-	2567 (56.8)
Salt intake (*n*, %)			<0.01	
High salt	412 (33.5)	1236 (37.6)	-	1648 (36.5)
Medium salt	633 (51.4)	1638 (49.8)	-	2271 (50.3)
Low salt	186 (15.1)	414 (12.6)	-	600 (13.2)
Educational level (*n*, %)			<0.01	
Primary/illiteracy	241 (19.6)	450 (13.7)	-	691 (15.3)
Middle school	815 (66.2)	2055 (62.5)	-	2870 (63.5)
University/college	175 (14.2)	783 (23.8)	-	958 (21.2)
Family income (¥/month)			<0.01	
<¥600	353 (28.7)	533 (16.2)	-	886 (19.6)
¥600 to ¥1000	457 (37.1)	1598 (48.6)	-	2055 (45.5)
≥¥1000	421 (34.2)	1157 (35.2)	-	578 (65.1)
Sleep quality	4.26 ± 3.16	3.55 ± 2.91	<0.01	3.74 ± 2.99
Good (PSQI score ≤ 3) (*n*, %)	364 (29.6)	1200 (36.5)	<0.01	1564 (34.6)
Moderate (3 < PSQI score ≤ 5) (*n*, %)	663 (53.9)	1664 (50.6)	-	2327 (51.5)
Poor (PSQI score > 5) (*n*, %)	204 (16.6)	424 (12.9)	-	628 (13.9)
Sleep duration (h)	6.74 ± 1.20	6.98 ± 1.48	<0.01	6.93 ± 1.4
Short (<7) (*n*, %)	484 (39.3)	707 (21.5)	<0.01	1191 (26.4)
Normal (7 ≤ sleep duration ≤ 8) (*n*, %)	600 (48.7)	2318 (70.5)	-	2918 (64.6)
Long (>8) (*n*, %)	147 (11.9)	263 (8.1)	-	410 (9.1)
Shift-work schedule (*n*, %)			<0.01	
Never	582 (47.3)	1509 (45.9)	-	2091 (46.3)
Occasional	341 (27.7)	1125 (34.2)	-	1466 (32.4)
Frequent	308 (24.9)	654 (19.9)	-	962 (21.3)

BMI, body mass index; SBP, systolic blood pressure; DBP, diastolic blood pressure; TC, total cholesterol; TG, total triglyceride; FBG, fasting blood glucose; PSQI, Pittsburgh Sleep Quality Index.

**Table 2:** Odds ratios (OR) with 95% confidence intervals of sleep quality, sleep duration and shift-work schedulefor the prevalence of hypertension in Chinese male adults.

Sleep Attributes	*n*	Unadjusted OR	Adjusted OR ^1^	Adjusted OR ^2^
Sleep quality (as continuous variable)	4519	1.08 (1.05–1.11) *	1.08 (1.05–1.11) *	1.07 (1.04–1.10) *
Sleep quality (as categorized variable)				
Good (PSQI score ≤ 3)	1564	(Reference)	(Reference)	(Reference)
Moderate (3 < PSQI score ≤ 5)	2327	1.33 (1.14–1.55) *	1.32 (1.13–1.55) *	1.22 (1.03–1.45) *
Poor (PSQI score > 5)	628	1.63 (1.32–2.02) *	1.74 (1.40–2.16) *	1.81 (1.44–2.29) *
Sleep duration (as categorized variable)				
Normal (7 h ≤ sleep duration ≤ 8 h)	2918	(Reference)	(Reference)	(Reference)
Short (<7 h)	1191	1.38 (1.20–1.58) *	1.40 (1.20–1.61) *	1.33 (1.13–1.56) *
Long (>8 h)	40	0.94 (0.65–1.34)	0.97 (0.67–1.4)	0.97 (0.66–1.41)
Shift-work schedule				
Never	2091	(Reference)	(Reference)	(Reference)
Occasional	1466	0.79 (0.67–0.92) *	0.79 (0.67–0.93) *	0.81 (0.68–0.96) *
Frequent	962	1.21 (1.02–1.44) *	1.23 (1.03–1.47) *	1.20 (1.01–1.44) *

Adjusted OR ^1^: adjust for age;Adjusted OR ^2^: adjust for age, BMI, TC, TG, FBG, physical activity, smoking, drinking, salt intake, educational level and family income; PSQI, Pittsburgh Sleep Quality Index. * *p* < 0.05.

**Table 3:** Odds ratios (OR) with 95% confidence intervals of different combinations of sleep quality, sleep duration and shift-work schedule for the prevalence of hypertensionin Chinese male adults.

Poor Sleep Quality	Short Sleep Duration	Occasional Shift-Work Schedule	Frequent Shift-Work Schedule	*n* (%)	Unadjusted OR	Adjusted OR ^1^	Adjusted OR ^2^
−	−	−	−	646 (14.3)	(Reference)	(Reference)	(Reference)
+	−	−	−	764 (16.9)	1.20 (0.93–1.54)	1.19 (0.92–1.54)	1.25 (0.96–1.63)
−	+	−	−	199 (4.4)	0.69 (0.45–1.06)	0.74 (0.48–1.14)	0.80 (0.51–1.24)
+	+	−	−	511 (11.3)	1.67 (1.28–2.19) *	1.65 (1.25–2.16) *	1.74 (1.31–2.31) *
−	−	+	−	322 (7.1)	0.70 (0.49–0.99) *	0.69 (0.49–0.98) *	0.67 (0.50–0.89) *
+	−	+	−	626 (13.9)	0.76 (0.58–1.01)	0.77 (0.58–1.02)	0.76 (0.57–1.02)
−	+	+	−	85 (1.9)	0.67 (0.37–1.24)	0.64 (0.34–1.19)	0.59 (0.30–1.14)
+	+	+	−	408 (9.0)	1.45 (1.08–1.93) *	1.45 (1.08–1.95) *	1.43 (1.05–1.95) *
−	−	−	+	245 (5.4)	1.24 (0.88–1.76)	1.30 (0.91–1.85)	1.33 (0.92–1.91)
+	−	−	+	403 (8.9)	1.37 (1.02–1.83) *	1.34 (0.97–1.85)	1.34 (0.98–1.80)
−	+	−	+	54 (1.2)	1.35 (0.71–2.57)	1.47 (0.77–2.82)	1.21 (0.60–2.46)
+	+	−	+	255 (5.6)	1.76 (1.27–2.45) *	1.89 (1.35–2.64) *	1.97 (1.40–2.79) *

Poor sleep quality: PSQI score > 5; Short sleep duration: sleep duration < 7 h; occasional shift-work schedule: no more than 1 times of night shift per week; frequent shift-work schedule: 2 or more times of night shift per week. Adjusted OR ^1^: adjust for age; Adjusted OR ^2^: adjust for age, BMI, TC, TG, FBG, physical activity, smoking, drinking, salt intake, family income and educational level. * *p* < 0.05.
